# Topical Prednisolone Acetate Challenge as a Predictor of Intraocular Pressure Elevation Following Ozurdex® in Diabetic Macular Edema: A Retrospective Study

**DOI:** 10.7759/cureus.92761

**Published:** 2025-09-19

**Authors:** Ahmed B Alsatrawi, Hasan B Alhaddar, Ali R Mubarak

**Affiliations:** 1 Ophthalmology, Salmaniya Medical Complex, Manama, BHR

**Keywords:** diabetic macular edema (dme), intraocular pressure (iop), iop, medical retina and glaucoma, ocular hypertension (oht), ozurdex, retina, steroid

## Abstract

Background

Steroid-induced ocular hypertension remains a practical concern with intravitreal corticosteroid therapy. The dexamethasone implant (Ozurdex®) is widely used for diabetic macular edema (DME), yet increases in intraocular pressure (IOP) can still occur. Identifying patients at greater risk before treatment would allow more tailored counseling and follow-up. This study examined whether a short topical prednisolone acetate challenge can anticipate the IOP response to Ozurdex in routine care within a Middle Eastern cohort.

Methods

This retrospective, observational study included 27 eyes of 27 patients with DME treated at Salmaniya Medical Complex, Kingdom of Bahrain. All patients underwent a topical prednisolone acetate 1% challenge (QID for three to four weeks) before Ozurdex implantation. IOP was measured at baseline, after the topical challenge, and six to eight weeks post-Ozurdex using Topcon non-contact tonometry. The primary outcome was a clinically significant IOP rise after Ozurdex (≥10 mmHg). Friedman and Wilcoxon signed-rank tests were used for within-eye comparisons; correlation was assessed with Spearman’s ρ.

Results

Mean baseline IOP was 18.4±2.2 mmHg, increasing to 19.6±2.5 mmHg after topical steroid challenge and 21.4±5.5 mmHg at six to eight weeks post-Ozurdex. Across the three timepoints, IOP differed significantly (χ²=14.47, p=0.0007). Pairwise comparisons confirmed significant rises from baseline to challenge (p=0.0034) and from baseline to Ozurdex (p=0.0033). The IOP change after topical challenge correlated moderately with post-Ozurdex IOP rise (Spearman’s ρ=0.52, p=0.005). Clinically significant IOP elevation (≥10 mmHg) occurred in two eyes (7.4%), all managed medically without surgery.

Conclusion

A short-term topical prednisolone acetate challenge provides moderate predictive value for identifying eyes at risk of IOP elevation following Ozurdex implantation. While not a perfect predictor, this inexpensive and non-invasive test may assist clinicians in tailoring post-injection monitoring strategies, particularly in resource-limited or high-volume care settings.

## Introduction

Steroid-induced ocular hypertension (OHT) is a well-documented clinical phenomenon. Landmark work by Armaly and Becker established that individuals vary widely in their intraocular pressure (IOP) response to topical glucocorticoids, with some demonstrating high responsiveness [1,2]. The underlying mechanism is thought to involve trabecular meshwork remodeling and extracellular matrix accumulation, leading to increased resistance to aqueous humor outflow [3,4].

In clinical practice, this concern extends to intravitreal steroid therapies such as the dexamethasone implant (Ozurdex®), which is widely used for diabetic macular edema (DME). Clinical trials, such as the pivotal MEAD study, and subsequent real-world series, have confirmed that IOP elevations are not uncommon, typically peaking within six to eight weeks of injection, though most are medically controlled [5-8]. The possibility of predicting which patients are at risk is appealing. While topical steroid provocation tests have shown promise in anticipating IOP rises after intravitreal triamcinolone [9], they have not previously been studied in the context of Ozurdex in our region. This retrospective study explores the predictive value of a topical prednisolone acetate challenge in a Bahraini DME population.

## Materials and methods

Study design and setting

This was a retrospective observational study conducted at the Department of Ophthalmology, Salmaniya Medical Complex, Kingdom of Bahrain, between December 2022 and November 2024. We included 27 eyes of 27 patients with DME who received a dexamethasone intravitreal implant (Ozurdex® 0.7 mg; Allergan/AbbVie) and completed a short topical steroid provocation test prior to implantation.

Case identification and eligibility criteria

Review of the ophthalmology department minor operating room registry at Salmaniya Medical Complex, where all Ozurdex injections are routinely logged, showed that 223 patients had received Ozurdex® (dexamethasone intravitreal implant, 0.7 mg; Allergan/AbbVie) during the two-year period. Their electronic medical records were subsequently screened to determine eligibility.

Patients were eligible if they had a diagnosis of DME, were treatment-naïve with respect to Ozurdex (no prior implant and no concurrent intravitreal corticosteroids), had undergone a short course of topical prednisolone acetate 1% (four times daily for three to four weeks) prior to implantation, and had complete IOP data available at all three predefined time points (baseline, post-challenge, and six to eight weeks after injection).

Exclusion criteria included incomplete IOP data, missed follow-up, prior incisional glaucoma surgery, active uveitis or angle-closure, or initiation of new topical steroids or IOP-lowering agents during the study period. Eyes on stable pre-existing glaucoma therapy at baseline were permitted if their regimen remained unchanged throughout follow-up. After applying these criteria, 27 eyes from 27 patients with DME were included in the final analysis.

Steroid provocation protocol

Prednisolone acetate 1% was instilled QID for three to four weeks. IOP was measured at baseline (pre-challenge) and at the end of the challenge (three to four weeks).

Ozurdex procedure and follow-up

Ozurdex (0.7 mg) was administered in standard aseptic fashion. IOP was measured again at six to eight weeks post-implant, the expected window for peak steroid-related IOP rise.

Measurements and variables

The primary variable of interest was IOP (measured in mmHg) recorded at three predefined timepoints: baseline, after completion of the topical steroid challenge, and six to eight weeks following Ozurdex implantation. Measurements were obtained using a Topcon non-contact tonometer (air-puff) as part of the routine clinic workflow, with each recorded value representing the device’s automated averaged output according to manufacturer protocol. Two derived variables were calculated to represent changes relative to baseline: the difference between post-challenge and baseline IOP (ΔIOP_challenge) and the difference between post-Ozurdex and baseline IOP (ΔIOP_Ozurdex). Demographic and clinical covariates included patient age, sex, laterality, and lens status (phakic or pseudophakic). Because non-contact tonometry can yield slightly higher readings than Goldmann applanation tonometry at higher pressures, the same device was consistently used across all visits, and interpretation focused on within-eye changes rather than cross-modality comparisons.

Outcomes

The primary outcome of the study was the development of a clinically significant rise in IOP following Ozurdex implantation, which was defined in advance as an increase of 10 mmHg or more relative to baseline. Secondary outcomes included the absolute IOP recorded at six to eight weeks after implantation, the proportion of patients who required initiation or escalation of IOP-lowering therapy during follow-up, and exploratory classification of steroid responsiveness. Exploratory categorization of steroid responsiveness was performed using thresholds originally described by Armaly and Becker, in which an IOP increase of more than 15 mmHg is considered a high response, an increase of 6 to 15 mmHg is considered a moderate response, and an increase of less than 6 mmHg is considered a low response [1,2].

Statistical analysis

All statistical tests were two-sided with a significance threshold of α=0.05. Given the sample size of 27 eyes and the possibility of non-normal data distribution, non-parametric methods were applied. Descriptive statistics were summarized as means with SDs, medians with interquartile ranges, or counts with percentages, as appropriate. IOP was compared across the three timepoints using the Friedman test, with pairwise differences assessed by the Wilcoxon signed-rank test and Holm-Bonferroni correction for multiple comparisons. The predictive performance of the topical steroid challenge was examined through correlation analysis using Spearman’s rank coefficient, discrimination analysis with receiver operating characteristic (ROC) curves, and regression modeling. The ROC analysis evaluated the ability of ΔIOP_challenge to predict a clinically significant IOP rise (ΔIOP_Ozurdex ≥10 mmHg), with area under the curve (AUC), CIs, and optimal cut-off values reported alongside sensitivity, specificity, positive predictive value, and negative predictive value. Logistic regression was planned to assess predictors of clinically significant IOP rise, with ΔIOP_challenge as the primary covariate, and baseline IOP and lens status considered if event counts permitted; in the event of fewer observed cases, exact odds ratios (OR) with mid-P CIs were reported. Exploratory subgroup analyses were conducted according to lens status (phakic versus pseudophakic) and age stratified by median split. Complete-case analysis was used, as eyes with missing IOP data were excluded by design. All analyses were performed using SPSS version 29 (IBM Corp., Armonk, NY, USA) or R version 4.x.

Ethics

This study was conducted in accordance with the Declaration of Helsinki and approved by the Research & Ethics Committee, Salmaniya Medical Complex (Kingdom of Bahrain), with a waiver of informed consent due to its retrospective design and de-identified data handling.

## Results

Patient characteristics

A total of 27 eyes from 27 patients with DME were included in the analysis. The mean age of the cohort was 65.1±6.1 years, with an age range of 52 to 79 years. Fourteen patients (51.9%) were female, and 13 (48.1%) were male. With respect to lens status, 19 eyes (70.4%) were phakic and eight eyes (29.6%) were pseudophakic.

Intraocular pressure changes

The mean baseline IOP was 18.4±2.2 mmHg, increasing to 19.6±2.5 mmHg following the topical prednisolone acetate challenge, and further to 21.4±5.5 mmHg at six to eight weeks post-Ozurdex implantation (Table 1).

**Table 1:** IOP across study timepoints (n=27 eyes) Values are presented as mean ± SD, median, and range. IOP was measured at baseline, after a three to four-week topical prednisolone acetate challenge, and six to eight weeks post-Ozurdex implantation. IOP, intraocular pressure

Timepoint	Mean IOP (mmHg)	SD	Median	Range
Baseline	18.4	2.2	19	13-22
Post-steroid challenge	19.6	2.5	20	15-24
6-8 weeks post-Ozurdex	21.4	5.5	20	16-39

Within-eye comparison 

The Friedman test demonstrated a significant difference in IOP across the three study timepoints (χ²=14.47, p=0.0007). Post-hoc analysis with the Wilcoxon signed-rank test confirmed a significant increase from baseline to post-challenge (p=0.0034) and from baseline to post-Ozurdex (p=0.0033). A smaller but still statistically significant rise was also observed between the post-challenge and post-Ozurdex measurements (p=0.0475) (Table 2).

**Table 2:** Pairwise Wilcoxon signed-rank tests for IOP changes (n=27 eyes) Results of post-hoc comparisons between baseline, post-challenge, and six to eight weeks after Ozurdex implantation. Negative Z-values indicate the direction of change, and p-values were adjusted using the Holm-Bonferroni method. IOP, intraocular pressure

Comparison	Z-value	p-value
Baseline versus post-challenge	-2.92	0.0034
Baseline versus 6-8 weeks	-2.93	0.0033
Post-challenge versus 6-8 weeks	-1.99	0.0475

Steroid challenge as a predictor

**Figure 1:**
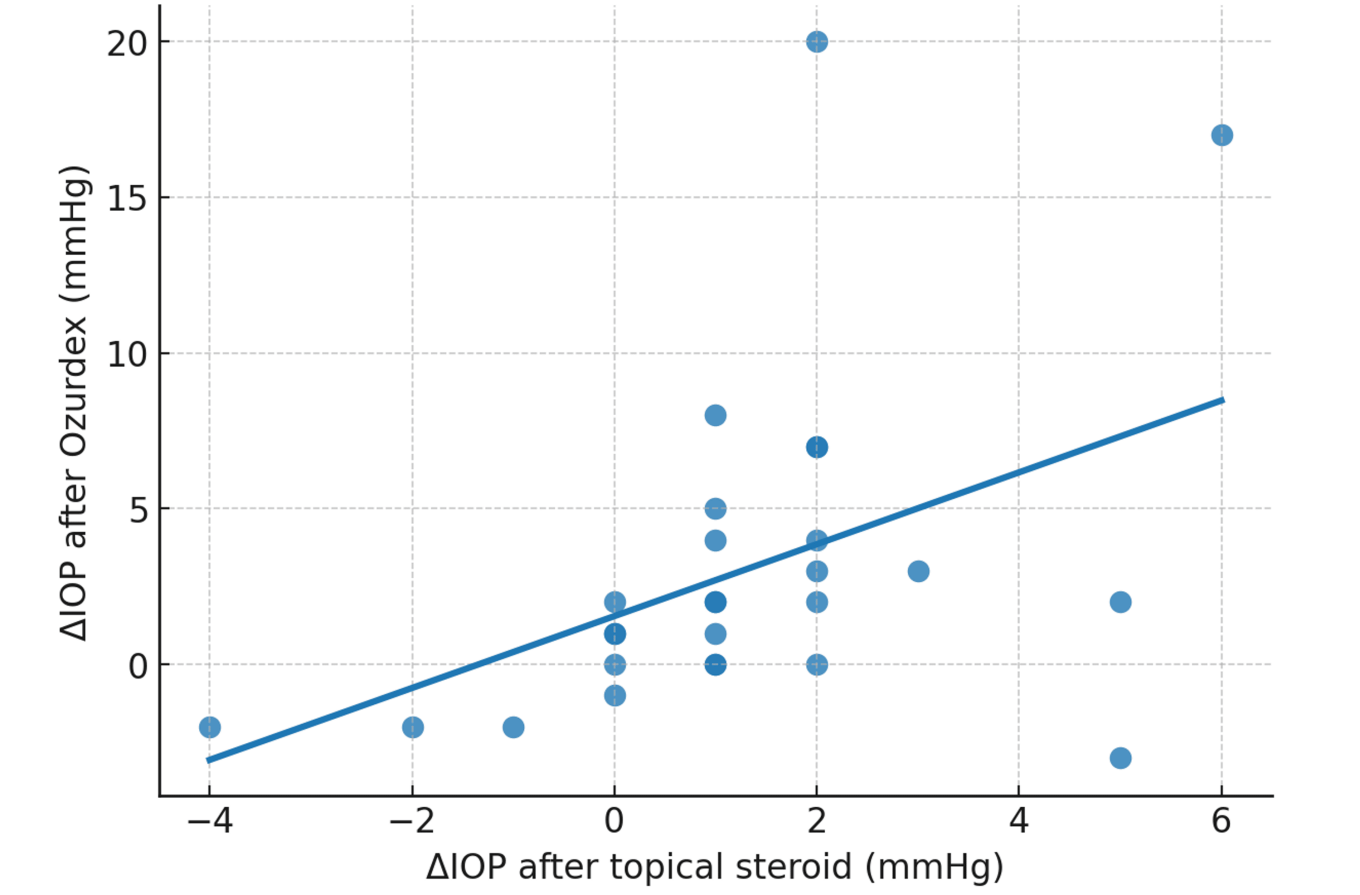
Correlation between ΔIOP after topical steroid challenge and ΔIOP after Ozurdex (n=27) Scatterplot showing the relationship between the change in IOP after the topical challenge (x-axis) and the change in IOP after Ozurdex implantation (y-axis). The regression line demonstrates a moderate positive correlation (ρ=0.52, p=0.005).

The IOP rise following the topical challenge (ΔIOP_challenge) showed a significant positive correlation with the IOP rise after Ozurdex (ΔIOP_Ozurdex) (Spearman’s ρ=0.52, p=0.005), indicating that patients with greater IOP response to topical steroids were more likely to exhibit higher IOP after Ozurdex (Figure 1).

Predictive value of the steroid challenge

**Figure 2:**
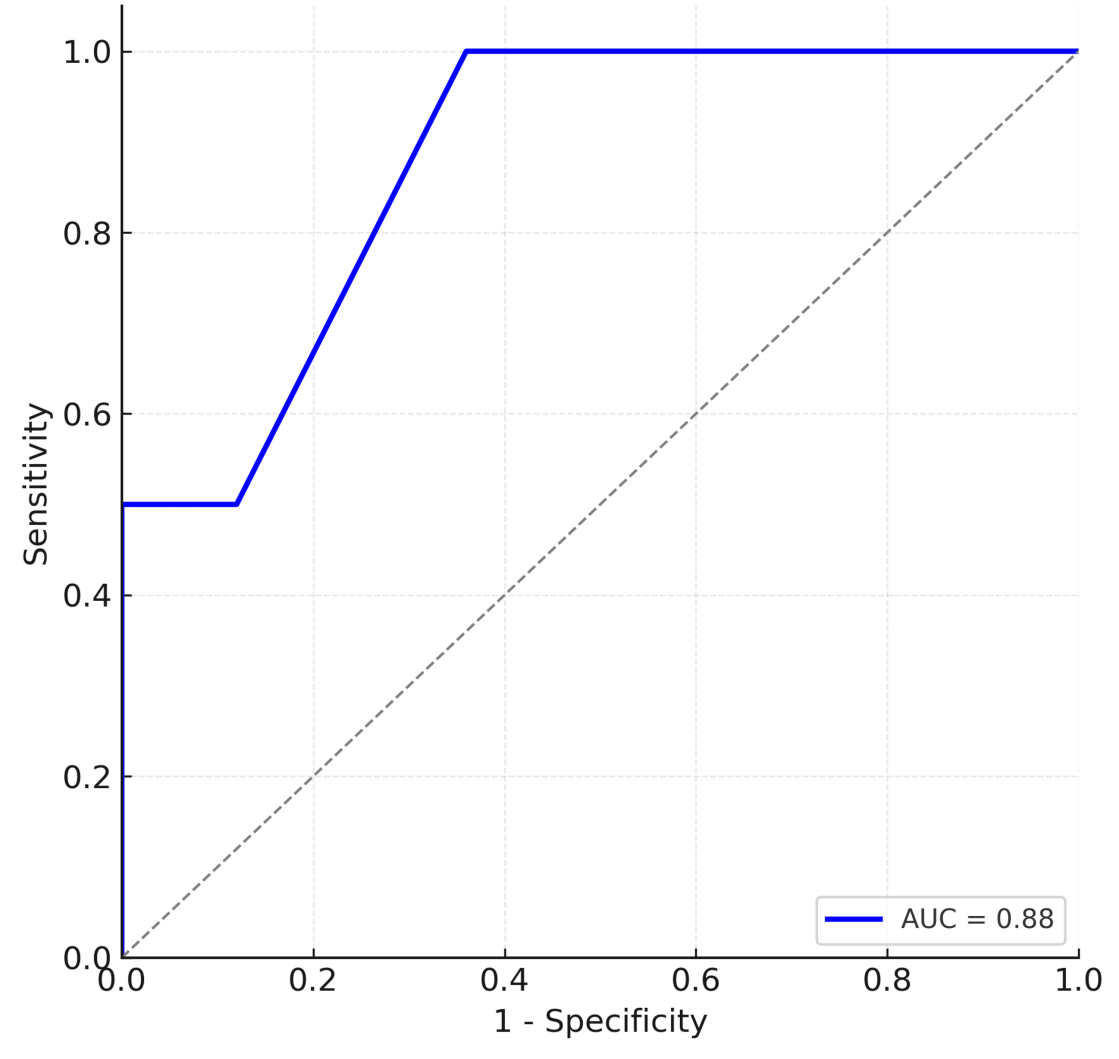
ROC curve for ΔIOP_challenge predicting ≥10 mmHg IOP rise after Ozurdex implantation The curve demonstrates the ability of the change in IOP after the topical steroid challenge to discriminate clinically significant IOP elevation following Ozurdex. The AUC was 0.88, indicating good predictive performance. IOP, intraocular pressure; ROC, receiver operating characteristic; AUC, area under the curve

ROC analysis further evaluated the discriminatory performance of the topical challenge for predicting a clinically significant IOP rise. The ROC curve demonstrated strong predictive value, with an AUC of 0.88. The ROC plot is presented in Figure 2.

Regression analysis

Logistic regression modeling was performed to explore predictors of a clinically significant IOP rise of 10 mmHg or more following Ozurdex implantation. The analysis included ΔIOP_challenge and baseline IOP as covariates. A greater change in IOP during the topical challenge was associated with increased odds of a subsequent clinically significant rise after Ozurdex (OR=2.12, 95% CI=0.93-4.86, p=0.074). Baseline IOP did not demonstrate an independent association with risk (OR=0.99, 95% CI=0.38-2.59, p=0.980). These results are summarized in Table 3.

**Table 3:** Logistic regression predicting ≥10 mmHg IOP rise after Ozurdex (n=27 eyes) OR with 95% CI are presented for ΔIOP_challenge and baseline IOP as predictors. The small number of events (n=2) resulted in wide CIs; therefore, findings should be interpreted as exploratory. IOP, intraocular pressure; OR, odds ratio

Predictor	OR (95% CI)	p-value
ΔIOP_challenge	2.12 (0.93-4.86)	0.074
Baseline IOP	0.99 (0.38-2.59)	0.98

Clinical outcomes

At six to eight weeks following Ozurdex implantation, a clinically significant IOP rise of 10 mmHg or more relative to baseline was observed in two eyes (7.4%). Both cases were managed successfully with escalation of topical IOP-lowering therapy, and no patient required surgical intervention. The mean absolute IOP at this time point was 21.4±5.5 mmHg.

When patients were stratified according to steroid responsiveness thresholds, 22 eyes (81.5%) were classified as low responders, three eyes (11.1%) as moderate responders, and two eyes (7.4%) as high responders. The two high responders corresponded to the cases that demonstrated clinically significant IOP elevation and required additional topical therapy.

## Discussion

In clinical practice, one of the main concerns with Ozurdex® (dexamethasone intravitreal implant) is the potential for significant IOP elevation. In the pivotal MEAD study, approximately 15-16% of eyes experienced a rise of ≥10 mmHg, though the majority were successfully managed with topical medications [5,6]. Real-world evidence, such as the SAFODEX registry of 421 eyes, reported OHT in 28.5% of cases, while only 0.7% required glaucoma surgery [10]. Similarly, long-term follow-up, including data from the International Ozurdex Study Group, shows that IOP >25 mmHg occurs in ~26.5% of eyes receiving dexamethasone implants, while incisional glaucoma surgery is required in only ~0.5% of cases; most IOP rises are controlled medically [11-13]. This is consistent with our data, where no eye required surgical intervention during the observation period.

Our results highlight the potential predictive value of a topical steroid challenge test. We observed a moderate correlation (ρ=0.52, p=0.005) between IOP changes after prednisolone acetate drops and after Ozurdex injection. In addition to the correlation analysis, ROC evaluation demonstrated good discriminatory performance of the topical test, with an AUC of 0.88. Logistic regression analysis supported this trend, showing that greater IOP rise during the topical challenge was associated with increased odds of a subsequent clinically significant rise after Ozurdex (OR=2.12, 95% CI 0.93-4.86, p=0.074). Although the association did not reach statistical significance, likely due to the limited number of events, the directionality reinforces the potential role of the topical test in risk prediction. Taken together, these findings suggest that the short-term steroid challenge may be a clinically useful tool to identify eyes at greater risk of clinically significant IOP elevation after Ozurdex. This resonates with prior work on intravitreal triamcinolone, where Breusegem et al. reported that a topical dexamethasone test had 100% specificity and positive predictive value, though with limited sensitivity (25%) [12]. In other words, if a patient demonstrates an IOP rise with topical steroids, there is a high likelihood that they will also respond with elevated IOP after intravitreal steroid therapy.

Recent reports further support this concept, indicating that prior steroid responsiveness, regardless of the route of administration, is a key predictor for subsequent steroid-related OHT [14,15]. Our study builds upon these findings and is, to our knowledge, the first to apply this concept to Ozurdex implants specifically in a Middle Eastern cohort. When eyes were categorized according to classic responder thresholds, the majority were classified as low responders, while only two eyes (7.4%) demonstrated a high response. Importantly, these two cases corresponded to the clinically significant rises observed after Ozurdex. Both were managed successfully with topical IOP-lowering therapy, and no patient required surgical intervention. This real-world outcome reinforces earlier trial and registry data showing that steroid-induced IOP elevations are common but usually controllable with medical treatment.

Clinically, the topical steroid challenge is appealing: it is inexpensive, non-invasive, and easily implemented. A positive response can inform risk stratification, prompting closer IOP surveillance post-Ozurdex or steering clinicians toward alternative therapies in borderline cases. However, the predictive value is not absolute; in our study, only part of the variability in post-Ozurdex IOP was explained by the topical test. This underscores the multifactorial nature of steroid-induced IOP elevation, which involves trabecular meshwork physiology, genetic predisposition, and ocular comorbidities [3,4,16,17].

Strengths and limitations

Strengths of our study include the use of real-world data from a government hospital population, consistent IOP measurement at predefined timepoints, and inclusion of patients with stable glaucoma therapy. Limitations include the small sample size (n=27), reliance on non-contact tonometry rather than Goldmann applanation, and the variability of the topical challenge duration (three to four weeks). These factors may limit the generalizability of the findings.

## Conclusions

A short topical prednisolone acetate challenge may serve as a practical, low-cost tool to help identify eyes at higher risk of IOP elevation following Ozurdex implantation. In our series, although the mean IOP increased modestly, only a small proportion of eyes experienced clinically significant pressure rises, all of which were controlled medically without the need for surgery. The topical challenge, while not a perfect predictor, provides valuable risk stratification that can guide clinicians in tailoring post-injection monitoring strategies. In resource-limited or high-volume clinical settings, this simple test may support safer and more individualized care for patients receiving intravitreal corticosteroid therapy.
